# Piloting web-based structural competency modules among internal medicine residents and graduate students in public health

**DOI:** 10.3389/fpubh.2022.901523

**Published:** 2022-10-14

**Authors:** Max Jordan Nguemeni Tiako, Farah Rahman, Janice Sabin, Aba Black, Dowin Boatright, Inginia Genao

**Affiliations:** ^1^Department of Medicine, Brigham and Women's Hospital, Boston, MA, United States; ^2^Harvard Medical School, Boston, MA, United States; ^3^Loyola Stritch School of Medicine, Chicago, IL, United States; ^4^Department of Biomedical Informatics and Medical Education, University of Washington, Seattle, WA, United States; ^5^Department of Medicine, Yale School of Medicine, New Haven, CT, United States; ^6^Department of Emergency Medicine, New York University Grossman School of Medicine, New Haven, CT, United States

**Keywords:** graduate medical education, curriculum development, healthcare disparities, residency and internship, implicit bias training, racial bias

## Abstract

**Introduction:**

Fewer than half of internal medicine program directors report any health disparities curriculum. We piloted a web-based healthcare disparities module among internal medicine (IM) residents to test effectiveness and feasibility, compared to a convenient sample of graduate students enrolled in a public health equity course.

**Methods:**

IM residents participated in an in-person session (module 1: introduction to racial and ethnic health disparities), but first, they completed a pre-module knowledge quiz. Two weeks later, they completed module 2: “unconscious associations” and a post-module knowledge quiz. For the control arm Yale School of Public Health (YSPH) students enrolled in a course on health disparities completed the pre-module knowledge quiz, module 1, and 2 as required by their course instructor.

**Results:**

Forty-nine IM residents and 22 YSPH students completed the pre-module quiz and Module 1. The mean (SD) score out of 25 possible points for the IM residents on the pre-module quiz was 16.1/25 (2.8), and 16.6/25 (3.2) for YSPH students, with no statistically significant difference. Nineteen residents (38.8%) completed the post-module quiz with a mean score of 16.7/25 (2.2), Hedge's g =0.23, compared to 18 (81.8%) YSPH students, whose mean (SD) score was 19.5/25 (2.1), Hedge's g=1.05. YSPH students' post-module quiz average was statistically significantly higher than their pre-module test score, as well as the residents' post-module test (*P* < 0.001). In examining participants' responses to specific questions, we found that 51% (*n* = 25) of residents wrongly defined discrimination with an emphasis on attitudes and intent as opposed to actions and impact, compared to 22.7% (*n* = 5) YSPH students before the module, vs. 63.2% (*n* = 12) and 88.9% (*n* = 16) respectively after.

**Conclusion:**

After completing a healthcare disparities course, graduate students in public health saw greater gains in knowledge compared to IM residents. Residents' responses showed knowledge gaps such as understanding discrimination, and highlight growth opportunity in terms of health equity education. Furthermore, embedding health equity education in required curricular activities may be a more effective approach.

## Introduction

Racial and ethnic health disparities in the U.S. have been extensively documented. Between the Institute of Medicine's 2002 Unequal Treatment: Confronting Racial and Ethnic Disparities in Healthcare” (1) and the recent National Academy of Medicine's 2018 “National Healthcare Disparities Report,” evidence points toward a persistence of disparities. This especially exists for poor and uninsured populations, who are disproportionately from racial and ethnic minority backgrounds. It has been well-established that structural racism is a fundamental cause of health inequities in the U.S., (2) and still, social structures that shape and enable inequality are often rendered invisible in medical education. To that effect, scholars have called for greater inclusion of health equity and structural competency education in medical curricula (3). Structural competency is a framework aiming to highlight structural forces, including web of interpersonal networks, and environmental, political and socioeconomic factors that surround clinical encounters in order for healthcare professionals and trainees to better understand the conversations that take place there within and better serve their patients (4).

Residents are in a unique position when it comes to the issue of structural competency education: residency is meant to shape how they will practice medicine once independent, but during residency, competencies are vital not only to patient care, but also to residents as teachers of medical students. In addition to the aforementioned calls for greater structural competency education in graduate and undergraduate medical education, evidence of persistent health disparities has influenced key structures in graduate medical education, as reflected in the Health Quality Pathways of the Clinical Learning Environment Review (CLER) and required Accreditation Council for Graduate Medical Education (ACGME) competencies (5). Still, medical residents do not feel prepared to care for marginalized patient populations, as shown in multiple studies where the majority of internal medicine residents did not feel confident in their knowledge related to health disparities (6, 7). Residents who have undergone structural competency report that the training had a positive impact on their clinical practice and relationship with patients (8). Still, the majority of internal medicine residency curricula do not include health disparities as a topic (9).

Along with recommendations for health disparities instruction in medical education, resources exist through the medical education portal of the American Association of Medical Colleges (AAMC), including the 2014's “Healthcare Disparities” (10) aimed at increasing residents' awareness of existing health disparities and their comfort level to improve their approach to patient care. As an interactive, web-based application course, this may be an ideal tool to educate residents about health inequities while allowing them to learn in a self-directed manner, as evidence shows that making equity-related trainings mandatory can have a negative impact on participants' attitudes, while autonomous motivation to participate is associated with improvement in attitudes (11). We piloted this course in an urban, tertiary health center internal medicine residency. We sought to determine the effectiveness and feasibility of administering this course in an internal medicine residency by piloting it with a sample of residents and comparing them with a convenient sample of graduate students in public health enrolled in a health equity course. Our outcome measure was increase in knowledge of health disparities.

## Methods

### Study type

This is a pilot study comparing the effectiveness of an educational intervention, with internal medicine residents as the target population, and graduate students in public health as a convenient comparator sample.

### Study design

Participants were selected from the internal medicine residency program at the Yale School medicine/Yale New Haven Hospital, and the Yale School of Public Health, Masters level Health Disparities course during April and May 2019. Participants from both groups were given the chance to engage with an interactive e-learning course titled *Health Disparities* (10). The three-part course focuses on (1) Introduction to Racial & Ethnic Disparities in Healthcare (2) Unconscious Associations (3) Patient-centered Communication. The goal of the course is to educate users on the existence of racial and ethnic disparities in the United States, increase the awareness of their personal implicit biases, and foster active behavior changes to limit the effect these biases have on day to day interpersonal interactions. This course serves as an introduction to structural competency for learners in that it emphasizes the ways in which societal structures and individual attitudes and behaviors can affect health outcomes, especially for marginalized populations.

Prior to starting the interactive modules, all participants were asked to take an assessment titled “pre-module quiz” to assess their baseline level of knowledge regarding health disparities. The quiz was administered on google forms, and all users were assigned an anonymous participant code based. The questions on the quiz were based on the content in the health equity course, and previously validated in a national sample of physicians (see appendix 1). Once participants had completed the three-part course, they were instructed to take the “post-module quiz” by entering the same unique identifier. The “post module quiz” was a re-administration of the same questions presented in the pre-module quiz. The outcome of this intervention, growth in health disparity knowledge, was based on the change in score between the pre-module and post-module quiz.

The intervention was conducted in two phases for both groups. Phase 1 was administered in person in a group setting. Participants completed the pre-module quiz and module (1) Introduction to Racial & Ethnic Disparities in Healthcare. After a two-week period, participants were contacted to complete Phase 2 which consisted of completing module (2) “Unconscious Associations” and the post module quiz. Module (3) Patient-centered Communication was optional. To encourage full completion of the intervention, residents were offered $25 gift card as an incentive.

Our comparison group is a cohort of Masters in Public Health (MPH) students at the Yale School of Public Health (YSPH) enrolled in ‘Health Disparities,' a course dedicated to understanding a wide array of health disparities and social determinants of health in the United States. To maintain consistency with resident physician group, Phase 1 was completed in a group setting, consisting of the pre-module quiz and module (1). Phase 2 consisting of module 2 and the post quiz were required, by the course instructor, to be completed after a 2-week period over spring break. There was no financial incentive attached to YSPH students, instead it was a course requirement. There was no grade associated with the completion of the quiz for classroom purposes. The study was approved by the Yale Institutional Review board (IRB 2000023797).

### Outcomes

Outcomes of interest were completion of the second module, and improvement in health disparities knowledge as measured by our pre- and post-module knowledge quiz.

### Statistical methods

Primary data points collected in this study are the answers to the quiz questions. The only demographics collected within this study were the unique participant identifier for cross comparison and group affiliation, resident or MPH student. Descriptive statistics were used to examine the difference in baseline knowledge, as well as knowledge improvement after participating in the pilot. Hedge's g scores (a measure of effect size which tells us how much one group differs from another) were used to assess changes in scores before and after the pilot. Analyses were performed using Stata version 16.1 (Statacorp LLC, College station, TX). All statistical tests were two-tailed and P < 0.05 was considered statistically significant.

## Results

Forty-nine internal medicine residents completed the pre-module quiz and Module 1, 53% (26) were women, and 47% (23) were men. Twenty-two public health students completed the pre-module quiz and module 1, 86.4% (19) were women and 13.6% (3) were men. The mean (SD) scores of participants pre-module quiz were 16.1/25 (2.8) for IM residents and 16.6/25 (3.2) for YSPH students. We found no statistically significant difference among the groups in participant's pre-module quiz scores.

**Figure 1:**
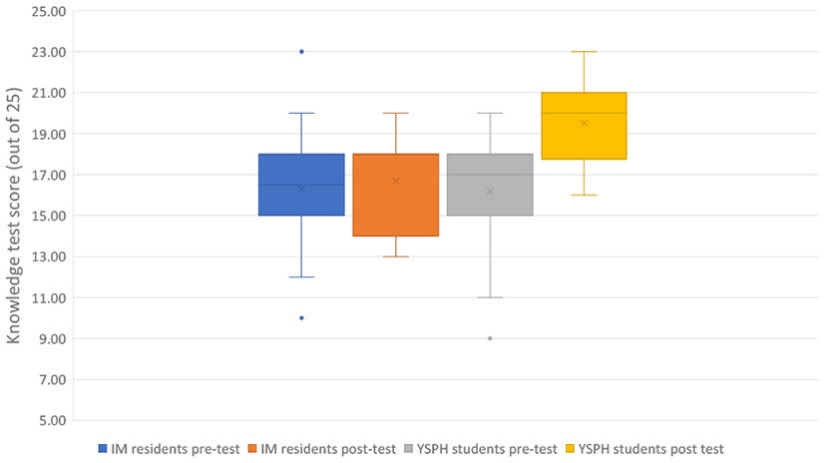
Average test score between internal medicine (IM) residents and Yale School of Public Health (YSPH) students before and after the course.

Nineteen residents (38.8%) completed the post-module quiz with a mean score of 16.7/25 (2.2), Hedge's g =0.23. In the comparison arm, 18 (81.8%) participants took the post-module quiz. Their mean (SD) score was 19.5/25 (2.1), Hedge's g = 1.05. YSPH students' post-module quiz average was significantly higher than the resident's (*P* < 0.001) (see Figure 1). The attrition rate for residents for the 2-week follow up and post module quiz completion was 61.2%, compared to 18.2% for the YSPH students.

**Figure 2:**
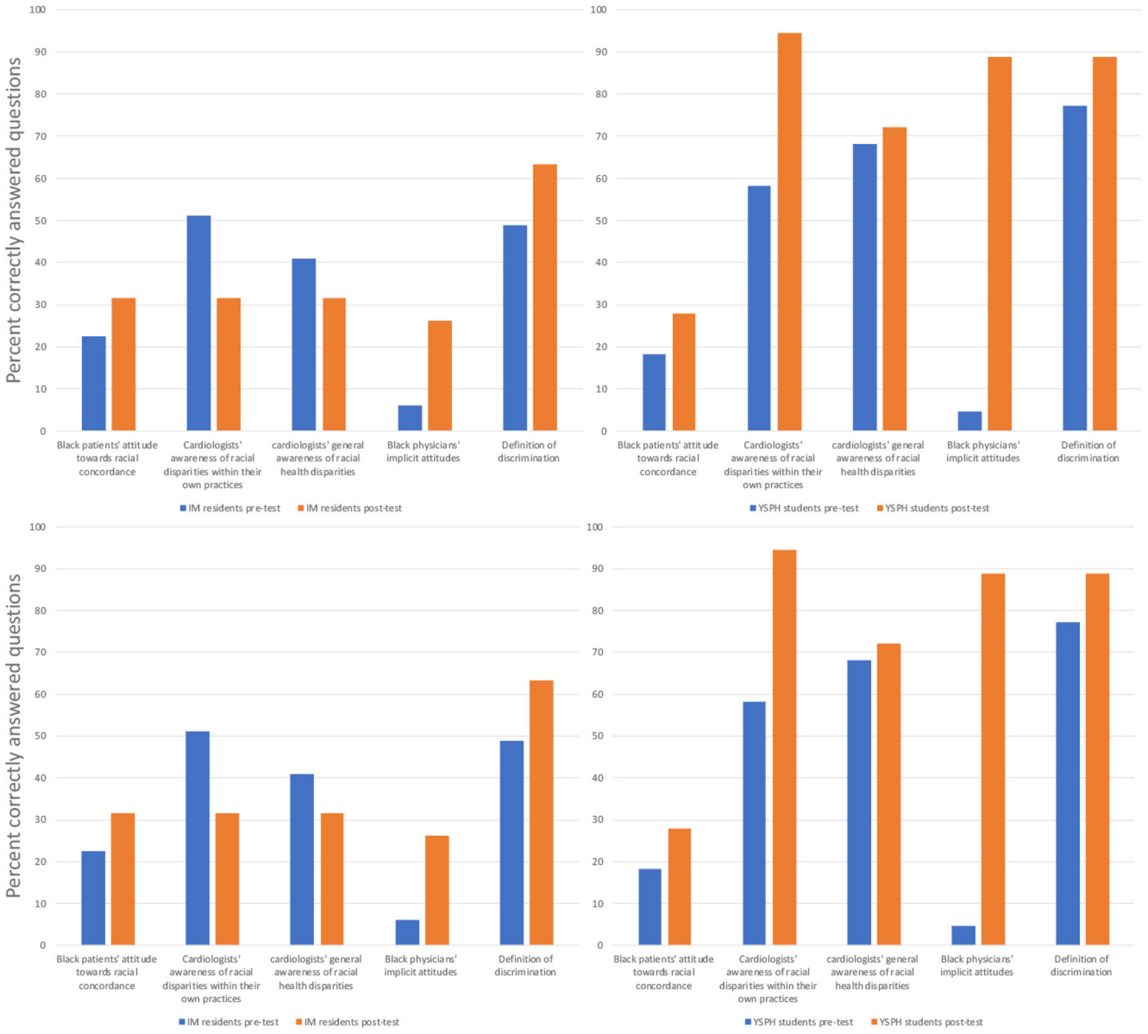
Comparing internal medicine (IM) residents to Yale School of Public Health (YSPH) students' average correct response rates before and after completing the healthcare disparities course.

We also examined participants responses to individual questions before and after the course (see Figure 2), and by group. First, in terms of the definition of discrimination, in the pre-test results, 51% (*n* = 25) of residents defined discrimination wrongly, selecting answers focused on intent and attitudes as opposed to actions and consequences, compared to only 22.7% (*n* = 5) of YSPH students (*P* = 0.03). After the module, 63.2% (*n* = 12) of residents defined discrimination correctly, improved from 49%, not statistically significant, as did 88.9% (*n* = 16) of YSPH students.

Second, when asked about Black physicians' implicit racial attitudes among physicians as reported in a previous large study (12), in the pre-test, 73% (*n* = 36) of residents wrongly answered that the majority of Black physicians held unconscious pro-White vs. Black bias, 18.8% (*n* = 9) answered that Black physicians held pro-Black vs. White bias, and 6.1% (*n* = 3) rightly answered that Black physicians in this sample held no implicit preferences, compared to 52.4% (*n* = 12), 18.2% (*n* = 4), and 4.5% (*n* = 1), respectively, among YSPH students. After taking the course, 26.3% (*n* = 5) of residents answered that Black physicians held no implicit preferences, compared to 88.9% (*n* = 16) of YSPH students.

Third, when asked about Black patients' attitudes toward physician-patient racial concordance, (13) in the pre-test, only 22.4% (*n* = 11) of residents rightly answered that Black patients report that physician's race is less important than whether the physician understood them, compared to 18.2% (*n* = 4) of YSPH students. After taking the course, 31.6% (*n* = 6) of residents answered this question correctly, as did 27.8% (*n* = 5) of YSPH students.

Fourth, in terms of physicians' awareness of health disparities (14), when asked about cardiologists' general awareness of racial healthcare disparities in the U.S., in the pre-test, 40.8% (*n* = 20) of residents correctly estimated cardiologists' awareness, whereas 44.9% (*n* = 22) overestimated their awareness, compared to, respectively, 68.2% (*n* = 15) and 9.1% (*n* = 2) of YSPH students. After taking the course, 31.6% (*n* = 6) of residents correctly estimated cardiologists' awareness, and 52.6% (*n* = 10) overestimated their awareness, compared to 72.2% (*n* = 13) and 5.6% (*n* = 1) among YSPH students. When asked about cardiologists' awareness of racial health disparities within their own patient population, 51.0% (*n* = 25) of residents correctly estimated their awareness, compared to 68.2% (*n* = 15) of YSPH students. After taking the course, however, only 31.6% (*n* = 6) of residents correctly estimated cardiologists' awareness, while 68.4% (*n* = 13) overestimated it, compared to 94.4% (=17) and 5.6% (*n* = 1) of YSPH students.

## Discussion

This online healthcare disparities course led to a significant increase in knowledge regarding health disparities among graduate students in a public health course, but not among internal medicine residents. A close look at the residents' vs. YSPH participants' responses to the quiz highlights important, remediable knowledge gaps as well as growth areas in physicians' education regarding health disparities. The attrition from participation in this pilot study was significantly higher among internal medicine residents compared to public health students, who were required to complete the course as part of their curriculum.

While many studies have evaluated internal medicine residents' knowledge following health equity courses, this is the first to our knowledge to compare their knowledge to that of graduate students in public health, who would ostensibly be, among graduate and professional trainees, some of the most aware of the social determinants of health and their downstream impacts on patient health. Given that the public health students were enrolled in an elective health disparities course, this group likely had a greater inclination toward furthering their knowledge on the subject. It is possible that the background knowledge acquired through this course contributed to helping them further consolidate the information from our pilot study. The relative stagnation in scores among internal medicine residents may be due to lack of consolidation in the setting of increased cognitive load and competing demands of residency (15). A previous study on medical residents' knowledge and attitudes regarding health disparities showed that residents' knowledge increased after didactic teaching, however, mostly interest in the subject predicted engagement with the course content (16).

Residents' specific responses to the knowledge test provide some insights into how physicians perceive racial health disparities. First, the emphasis of intent instead of action and consequences when defining discrimination, unlike public health students, is in line with previous research. Studies from the social psychology literature shows that people who are more often targets of discrimination prioritize harm over intent when defining discrimination compared to observers (17). Additionally, in terms of racial groups, a study showed that White people were primarily influenced by intent, while Black people were influenced by intent and harm, likely attributable to the disproportionality of experienced racial discrimination (18). This study also found that perspective-taking increased White participants judgment of both intent and harm. This has implications for how physicians interpret reported experiences of discrimination, from patients and colleagues alike, as well as evidence of healthcare disparities. If intent is what matters most, then, they may be more inclined to dismiss claims of discrimination without proof of malintent, even in the face of disparate outcomes. For instance, study of medical students who witnessed discrimination during their clinical rotations showed that a barrier to students acting to address them was students perceiving doctors as “good people” who provide disparate care unintentionally, leading to a normalization of disparities, while these experiences strengthened commitment to equity specifically among justice-oriented students (19). Similarly, the emphasis of intent may explain why minority trainees who experience discrimination from patients and colleagues alike report little support from colleagues and clinical supervisors (20, 21).

Residents' overestimation of cardiologists' awareness of healthcare disparities at large and within their own practices compared to YSPH students is consistent with evidence that medical trainees perceive doctors as “good people” who thus may be conscientious of such issues. There is also a parallelism with recent evidence that Americans overestimate racial progress, and underestimate racial inequality (22). This phenomenon mirrors findings from a social psychology study on reminders of racial inequality. Exposure to evidence of racial inequality had a paradoxical effect on participants who previously overestimated racial progress assessed the past as more unequal, justifying their belief in perceived present-day equality (23).

Participants' overestimation of Black patients valuing racial concordance in patient-provider encounters over having a physician who understands them raises a concern about the over-emphasis of racial concordance as a solution to health inequities. Certainly, some evidence shows that there are benefits to racial concordance (24), however, the results are mixed. Physicians assuming that Black patients necessarily prefer Black doctors may impact patient care. This assumption may unnecessarily contribute to non-Black providers' interracial contact anxiety (25), and shape clinical encounters negatively. Consequences of these assumptions may include physician behaviors such as using fewer words and less positive body language when seeing Black patients (26), which may have implications for patients' likelihood to further engage and follow physicians' advice. (24) Participants' perception of Black physicians' implicit attitudes as pro-White is counter to two studies that found that Black physicians held no implicit bias in either direction (12). Recent approaches aiming to mitigate the impact of stereotyping in the workplace through implicit bias training often highlight the pervasiveness of implicit biases across racial groups. However, such approaches have consequences in that individuals who are exposed to messaging about high prevalence of stereotyping and prejudice are more likely to treat others in stereotype-consistent ways than those who are exposed to messaging about low prevalence of stereotyping (27). Additionally, the attribution of discrimination primarily to implicit bias can inadvertently lead to a lack of accountability for perpetrators (28).

Taken together, residents' responses to the knowledge test in both the pre- and post-test phases, in comparison to YSPH students highlights potential growth areas in current approaches in educating physicians and trainees about health and healthcare disparities. Introducing fundamentals, like a shared understanding of the meaning of discrimination and its implications, and leveraging existing evidence in fields such as social psychology and sociology would be paramount to create effective educational interventions.

While most internal medicine residents rate the quality of the health equity training they receive as very good or excellent, most program directors do not (9). This leadership's awareness of the lackadaisical nature of institutional support for health equity training also highlights the magnitude of this gap in education. Scholars have advocated for protected curricular time, faculty development, hiring of faculty experts, and institutional support of resident projects aimed at tacking health disparities (29). Online modules focused on health equity such as the one used for this pilot have the potential of filling gaps in medical education, as previously shown by a curriculum introduced at a professional conference (30). Nevertheless, in order for all trainees to receive this education, integration of health equity as a core component of protected curricular time is necessary given the competing demands of residents' free time.

Our study has limitations. First, the residents who participated in this pilot volunteered, whereas public health students were required to participate as part of an ongoing class. The degree of attrition among internal medicine residents, compared to public health students may be related to competing and high demands of residency training, especially when asked to voluntarily complete self-directed, optional learning. In addition, those who volunteered may have greater interest in health equity education, and potentially more knowledge than those who didn't. However, the volunteers represent nearly/over half of residents typically present during residents' educational conferences at one of three sites. There may be a selection bias in studying a group of public health students who have self-selected in a higher-level health disparities course. Additionally, posing this study as a requirement impacted their level of educational commitment and time dedication, as seen in their score improvement. We did not collect information on age, race, ethnicity, or citizenship. Specifically, the lack of racial demographic data from participants, precludes us from knowing how participants' race may have impacted our findings. Knowledge and awareness of health disparities does not necessarily lead to changes in behavior, however, awareness is necessary in order for physicians to identify barriers to equitable care within health systems and their individual practices. Lastly, the lack of open-ended questions within our survey, prevents us from gathering narrative understanding of how our participants view structural and social stressors that affect health outcomes.

## Conclusion

In this pilot of a web-based health disparities curriculum, we compared volunteer internal medicine residents' knowledge and improvement after the modules to that of graduate level public health students and found that public health students', who were enrolled in health disparities education as part of their curriculum, knowledge increased significantly more than that of internal medicine residents. Residents' knowledge and responses provided some insights into knowledge gaps and important growth areas of health equity education for medical trainee curriculum, especially the necessity to rely on evidence from multiple fields, such as social psychology and medical sociology. Most notable, lastly, was the attrition rate among internal medicine residents compared to public health students for whom participation was embedded in existing coursework and protected curricular time. Our study highlights the need for greater integration of health equity education in existing curricula with protected time for internal medicine residents to show greater success.

## Data availability statement

The raw data supporting the conclusions of this article will be made available by the authors, without undue reservation.

## Ethics statement

The studies involving human participants were reviewed and approved by Yale University, School of Medicine. The patients/participants provided their written informed consent to participate in this study.

## Author contributions

MN and IG conceived the idea for the project, attained the funding, enrolled participants, conducted in-person sessions, and contributed to the manuscript. MN performed the statistical analysis and wrote the final version of the manuscript. FR contributed to the design of the project, conducted outreach, enrolled participants, coordinated with study participants, performed the statistical analysis, and significantly contributed to the first draft of the manuscript. JS, AB, DB, and IG contributed to the study design, data interpretation, and review of the manuscript. All authors contributed to the editing, revision, and approval of the final manuscript.

## Funding

Yale Center for Clinical Investigation Yale Department of Internal Medicine, Internal Grant.

## Conflict of interest

The authors declare that the research was conducted in the absence of any commercial or financial relationships that could be construed as a potential conflict of interest.

## Publisher's note

All claims expressed in this article are solely those of the authors and do not necessarily represent those of their affiliated organizations, or those of the publisher, the editors and the reviewers. Any product that may be evaluated in this article, or claim that may be made by its manufacturer, is not guaranteed or endorsed by the publisher.
